# Activation of Biomass-Derived Carbon Platelets for EDLC Symmetrical Devices

**DOI:** 10.3390/nano16150957

**Published:** 2026-08-04

**Authors:** Vediyappan Thirumal, Perumal Rajivgandhi, Alagan Sekar, Jinho Kim

**Affiliations:** 1 Department of Mechanical Engineering, Yeungnam University, Gyeongsan-si 38541, Gyeongbuk-do, Republic of Korea; 2 Department of Chemistry, Nehru Memorial College, Puthanampatti, Trichy 621 007, Tamilnadu, India

**Keywords:** biomass source, tamarind fruit, seed shell, activated carbon, supercapacitor, symmetric device

## Abstract

The sustainable bio-activated carbon platelets were synthesized from tamarind (*tamarind indicia*) fruit seed shells (TFSs) by a pyrolysis approach with an inert gas atmosphere. The carbonization process was carried out at 800 °C under an inert argon atmosphere, yielding both pure TFS-AC and chemically activated TFS-AC (KOH) carbon materials. Microscopic surface morphological analysis confirmed the formation of thin, interconnected porous carbon platelet nanosheets with enhanced surface structural uniformity. Raman spectroscopy revealed characteristic D- and G-bands, signifying the presence of graphitic domains and partial structural disorder. BET surface area analysis indicated a significant improvement from 48.54 m^2^/g in TFS-AC to 124.72 m^2^/g in TFS-AC (KOH), suggesting enhanced pore development and surface accessibility due to KOH activation. Electrochemical two-electrode performance was evaluated in symmetric device configurations using 3M KOH aqueous electrolyte. The TFS-AC (KOH) device exhibited a remarkable specific capacitance, which delivered 129.03 F/g at 0.5A/g, compared to the pure TFS-AC device. Electrochemical impedance spectroscopy (EIS) further confirmed low internal resistance and favorable ion transport. These findings confirm that KOH-activated TFS-derived carbon nanosheets have higher electrochemical stability, retaining 98.2% capacitance over 10,000 cycles. These results are promising electrode materials for high-performance supercapacitor applications, owing to their superior electrochemical symmetric device performance of bio-mass carbon Tamarind seed shell platelet nanosheets for future energy storage symmetric device applications.

## 1. Introduction

Carbonaceous materials primarily consist of carbon atoms and can exist in various allotropic forms. Among them, activated carbon (AC) represents a class of amorphous carbonaceous materials characterized by a highly porous structure, large internal surface area, good thermal stability, and notable electrical conductivity [1]. These materials encompass a wide range of carbon allotropes including amorphous carbon (such as coal), activated charcoal, graphite and diamond [2]. Activated carbon typically exhibits a partially crystalline structure similar to that of activated charcoal. In recent years, considerable attention has been directed toward the development of activated carbon from natural and renewable biomass sources, particularly agricultural bio-waste, due to their abundance, low cost and sustainability [3,4].

The synthesis of activated carbon from agricultural waste generally involves chemical activation and thermal pyrolysis processes conducted at temperatures between 600 and 1000 °C [5]. Common chemical activating agents include KOH, NaOH, H_3_PO_4_ and ZnCl_2_, with KOH being widely used due to its strong ability to create microporous structures in the carbon matrix [6,7]. These activating agents significantly influence the textural properties and porosity of the resulting carbon materials. Bio-derived activated carbons have gained increasing interest in a wide range of applications such as electrochemical energy storage devices including supercapacitors [8], rechargeable batteries [9,10,11], fuel cells [12,13], and water purification systems [14,15].

Ongoing research explores various biomass precursors and activation strategies to tailor the pore structures and enhance the electrochemical performance of the resulting carbon materials [16,17]. Notably, naturally occurring biomass such as orange peels, rice straws, and rice husks have been reported as cost-effective and eco-friendly precursors with inherent microstructured features and microporosity, making them suitable for lithium-ion storage and supercapacitor applications [18,19]. Compared to synthetic carbon nanomaterials, bio-derived carbons offer advantages such as low production costs, environmental compatibility, and scalability. Furthermore, their use in electric double-layer capacitors (EDLCs) is particularly beneficial due to their tunable pore structures, high electrical conductivity and excellent electrochemical and thermal stability [20,21,22,23].

In recent years, the conversion of renewable bio-waste into valuable porous carbon materials has gained considerable attention for energy storage applications, particularly due to their inherently high micro- and mesoporous structures, which facilitate efficient ionic transport and storage. Among various materials, carbon-based nanostructures such as carbon nanotubes (CNTs), reduced graphene oxide (RGO), mesoporous carbon, carbide-derived carbon, carbon nanoparticles, carbon quantum dots (CQDs) and activated carbons derived from natural bio-waste resources have emerged as prominent candidates for supercapacitor electrodes due to their exceptional electrical conductivity and surface characteristics [23,24,25]. In addition, several studies have explored metal oxide-based nanocomposites such as MnMoO_4_ nanorods anchored on graphene nanosheets and NiCo-layered double hydroxide (NiCo-LDH/rGO), which exhibit promising performance as supercapacitor materials owing to their synergistic redox behavior and layered morphology [23,24,25].

Among all these materials, bio-derived activated carbon holds significant promise due to its low cost, environmental sustainability, and high surface area. Typically, the synthesis of activated carbon involves two major steps: (1) chemical activation using dilute acids or alkalis (e.g., H_3_PO_4_, KOH) and (2) thermal treatment or pyrolysis under controlled conditions in a muffle or tubular furnace [26,27]. Pyrolysis under inert gas atmospheres such as argon (Ar) or nitrogen (N_2_) is a widely employed method, as these noble gases provide a stable and non-reactive environment that facilitates the carbonization of biomass without unwanted oxidation reactions [26,27]. In contrast, pyrolysis in the absence of inert gas often leads to the formation of fly ash or incomplete decomposition of the bio-waste precursors [28].

Activated carbon materials derived from such processes typically exhibit specific capacitances ranging from 50 to 200 F/g, depending on their surface properties and pore structure. However, bio-derived activated carbons prepared through optimized pyrolysis and chemical activation of KOH, achieve much higher specific capacitances, often in the range of 100 to 600 F/g [29]. This enhanced performance is attributed to their large specific surface area, well-distributed pore volume, and excellent surface electrochemical activity that promotes effective ion adsorption/desorption during charge–discharge cycles. Furthermore, the chemical activation process using KOH at elevated temperatures induces pore formation and structural modification, ultimately leading to highly efficient electric double-layer capacitor (EDLC) behavior in the resulting carbon material [30,31].

Tamarind (*Tamarindus indica* L.) is an abundant agricultural biomass resource widely distributed throughout many Asian countries, particularly India, Thailand, Bangladesh, Sri Lanka and Indonesia, and is also cultivated in several African and South American regions [32]. The undecorticated tamarind seed coat contains relatively high amounts of cellulose and hemicellulose, while lignin is present in a comparatively lower proportion, making it a favorable lignocellulosic precursor for the production of porous carbon materials because of its high carbon yield and thermal stability [33,34]. During recent years, carbon materials prepared from tamarind seed coats and fruit shells have attracted considerable attention for electrochemical energy storage owing to their naturally developed porous structure, renewable origin, and low processing cost. Activated carbons derived from tamarind biomass have demonstrated promising electrochemical performance as supercapacitor electrodes, where an ionic liquid electrolyte enabled a specific capacitance of approximately 78 F/g at a current density of 0.5 A/g [35]. Furthermore, tamarind-derived porous carbons have also been investigated as efficient anode materials for rechargeable lithium-ion and sodium-ion batteries because their hierarchical pore structure facilitates electrolyte penetration, rapid ion diffusion, and structural stability during repeated charge–discharge cycles [36,37]. These characteristics suggest that tamarind fruit seed shells are a sustainable and economically attractive biomass precursor for developing high-performance porous carbon electrodes for advanced electrochemical energy storage devices.

In this work, we report the conversion tamarind fruit seed shells bio-waste into highly microporous platelets carbon sheets through pyrolysis using a tubular furnace under an argon (Ar) inert atmosphere at 800 °C. The resulting carbon exhibits a sheet-like morphology with an interconnected porous structure. Two types of electrode materials were investigated: pure activated carbon (TFS-AC) and chemically activated carbon using potassium hydroxide (TFS-AC (KOH)). These materials were evaluated as active electrodes analyzed in three-electrode configurations and symmetric supercapacitor devices to explore their electric double-layer capacitor (EDLC) performance.

## 2. Materials and Methods

### 2.1. Collection and Pre-Treatment of Tamarind Seed Shells

Tamarind fruit seed shells (TFSs), used as the carbon precursor, were collected from tamarind trees in South India (Tamil Nadu, Dharmapuri, Muthanur). The tamarind shells were manually removed from the fruits, and the seeds were sun-dried for one day. The dried seeds were then roasted over a medium flame using a household kitchen pan until the outer seed shell turned dark brown to black. After roasting, the brittle outer skin was manually peeled off and broken into small fragments. These were thoroughly washed with deionized (DI) water to remove surface impurities and dried in a hot-air oven at 100 °C for approximately 6 h. The dried TFSs were then ground into a fine powder using a domestic mixer. The total mass of the powdered sample was about 6 g, which was then divided equally for two pathways: (i) direct pyrolysis to obtain Tamarind seed-based activated carbon (TFS-AC) and (ii) chemical activation using potassium hydroxide (TFS-AC (KOH)).

### 2.2. Chemical Activation Using Potassium Hydroxide (KOH)

For chemical activation, 3 g of TFS powder was mixed with a 3M aqueous KOH solution in a 1:5 weight ratio (TFS: KOH). The mixture was stirred continuously until a homogeneous, reddish-brown gel-like paste formed. The resulting mixture was filtered and dried in an oven at 100 °C for 12 h. The dried KOH-treated TFS sample, exhibiting a dark brown color, was then subjected to pyrolysis in a chemical vapor deposition (CVD) tubular furnace.

### 2.3. Synthesis of Activated Carbon Materials

**Figure 1:**
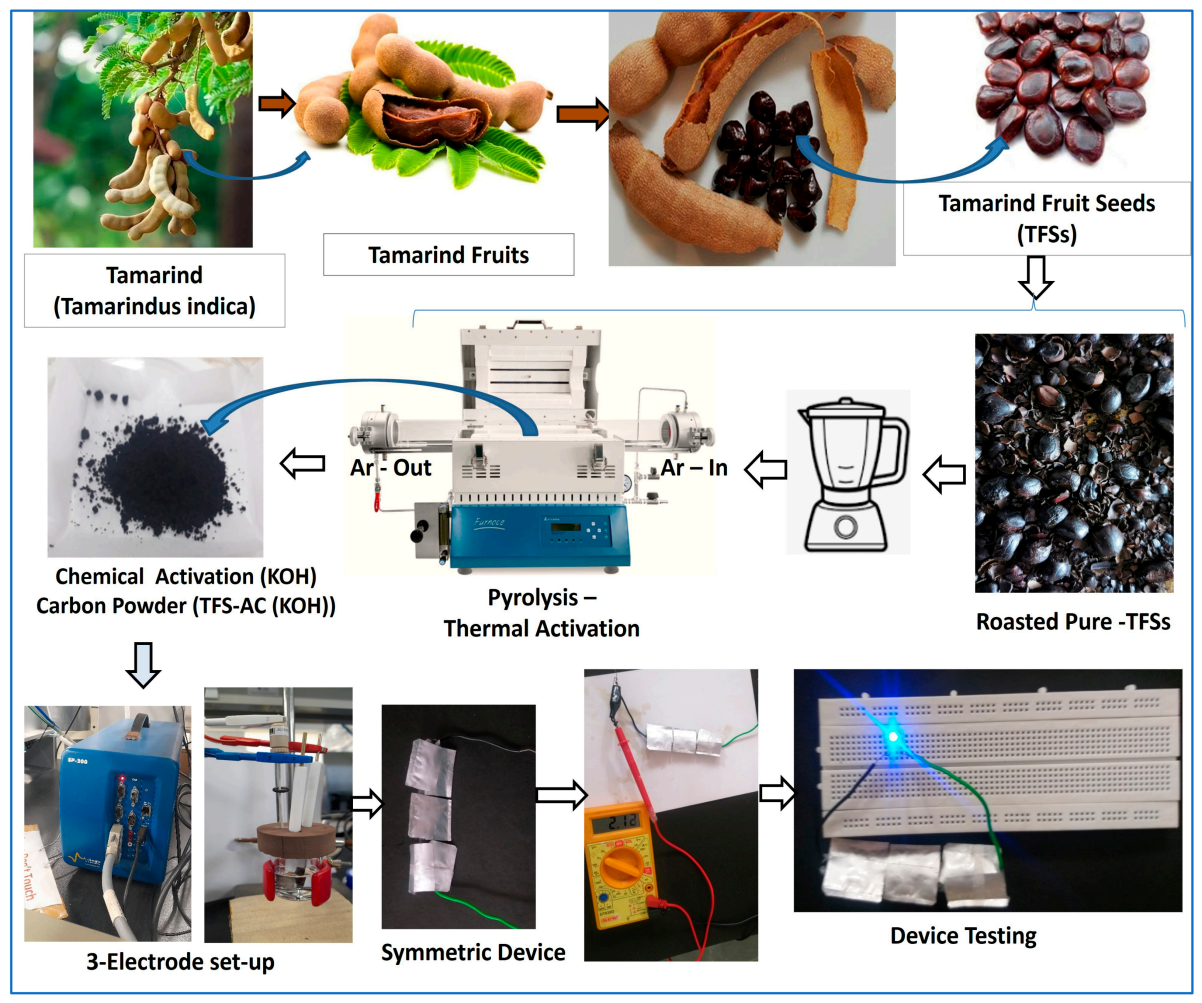
Schematic illustration of the experimental setup used for the carbonization process along with photographs of tamarind fruit seed shell (TFSs), chemical-treated TFSs and the electrochemical symmetric device along with an LED illumination demonstration confirming device performance.

Both untreated (pure TFSs) and chemically activated (TFS-KOH) powders were subjected to pyrolysis using a three-zone horizontal CVD tubular furnace (as shown in Figure 1). The powders were placed in ceramic boats and inserted at the center of the quartz tube in the furnace. The pyrolysis was carried out under an inert argon (Ar) atmosphere at a flow rate of 100 sccm. The furnace temperature was ramped to 800 °C at a rate of 5 °C/min and maintained for 2 h and 30 min. After completion, the furnace was allowed to cool naturally to room temperature under continuous Ar flow. The obtained carbonized powders were first washed with diluted hydrochloric acid (HCl) to remove residual inorganic impurities and amorphous carbon. Additionally, pure activated carbon from tamarind fruit seed shells (TFSs) was prepared using the same pyrolysis method without KOH activation. Subsequently, they were washed with DI water until the pH reached neutral (~7). The final carbon materials were designated as TFS-AC for pure pyrolyzed tamarind carbon, and TFS-AC(KOH) for KOH-activated carbon. These two samples were used for comparative analysis in subsequent characterization and electrochemical performance evaluations.

### 2.4. Materials Characterization

The as-prepared carbon-based electrode active materials, TFS-AC and TFS-AC (KOH), were systematically characterized to confirm their structural, morphological, and chemical properties. The surface morphology and porous network of the samples were observed using Field Emission Scanning Electron Microscopy (SEM, Quanta FEG-250) equipped (Hillsboro, OR, USA) with Energy Dispersive X-ray Analysis (EDAX) for elemental composition. Field Emission Transmission Electron Microscopy (TEM, Tecnai G2 T20) (Hillsboro, OR, USA) was employed to investigate the nanosheet thickness and pore architecture at higher resolution. Micro-Raman spectroscopy, manufacturer (Model) Horiba (XploRA) from (Palaiseau, France) using a 530 nm laser excitation source was used to evaluate the degree of graphitization and structural disorder via D- and G-band intensities. The surface area, pore size distribution, and total pore volume were analyzed using Physisorption analyzer, Brunauer–Emmett–Teller (BET) nitrogen adsorption–desorption measurements Manufacturer (Model) Micromeritics (3-FLEX) Norcross, GA, USA. X-ray photoelectron spectroscopy (XPS, PHI Versa-Probe III, Chigasaki, Japan), was performed to determine the surface chemical states and elemental bonding. Together, these techniques provided comprehensive insight into the effectiveness of KOH activation and its influence on porous architecture and electrochemical behavior.

### 2.5. Electrochemical Measurement

#### Three-Electrode Cell Setup

The electrochemical properties of the TFS-AC and TFS-AC (KOH) materials were first evaluated using a conventional system with a Bio-Logic SP-200 electrochemical workstation. The working electrodes were prepared by mixing the active material (TFS-AC or TFS-AC (KOH)), conductive carbon black, and polyvinylidene fluoride (PVDF) in an 80:10:10 weight ratio using N-methyl-2-pyrrolidone (NMP) as the solvent. The resulting slurry was coated uniformly on a pre-cleaned nickel foam substrate and dried in an oven at 100 °C for 5 h. The prepared nickel foam electrodes were then used as the working electrodes. A silver/silver chloride (Ag/AgCl) electrode (in saturated KCl) served as the reference electrode and a platinum (Pt) wire was used as the counter electrode. All measurements were carried out in a 3 M KOH aqueous electrolyte. Electrochemical studies included cyclic voltammetry (CV) in the scan rate range of 10 to 100 mV/s, galvanostatic charge–discharge (GCD) tests at various current densities from 1 to 10 A/g, and electrochemical impedance spectroscopy (EIS) in the frequency range of 100 kHz to 1 Hz with an AC amplitude of 10 mV.

## 3. Results and Discussion

### 3.1. X-Ray Diffraction (XRD) Analysis

**Figure 2:**
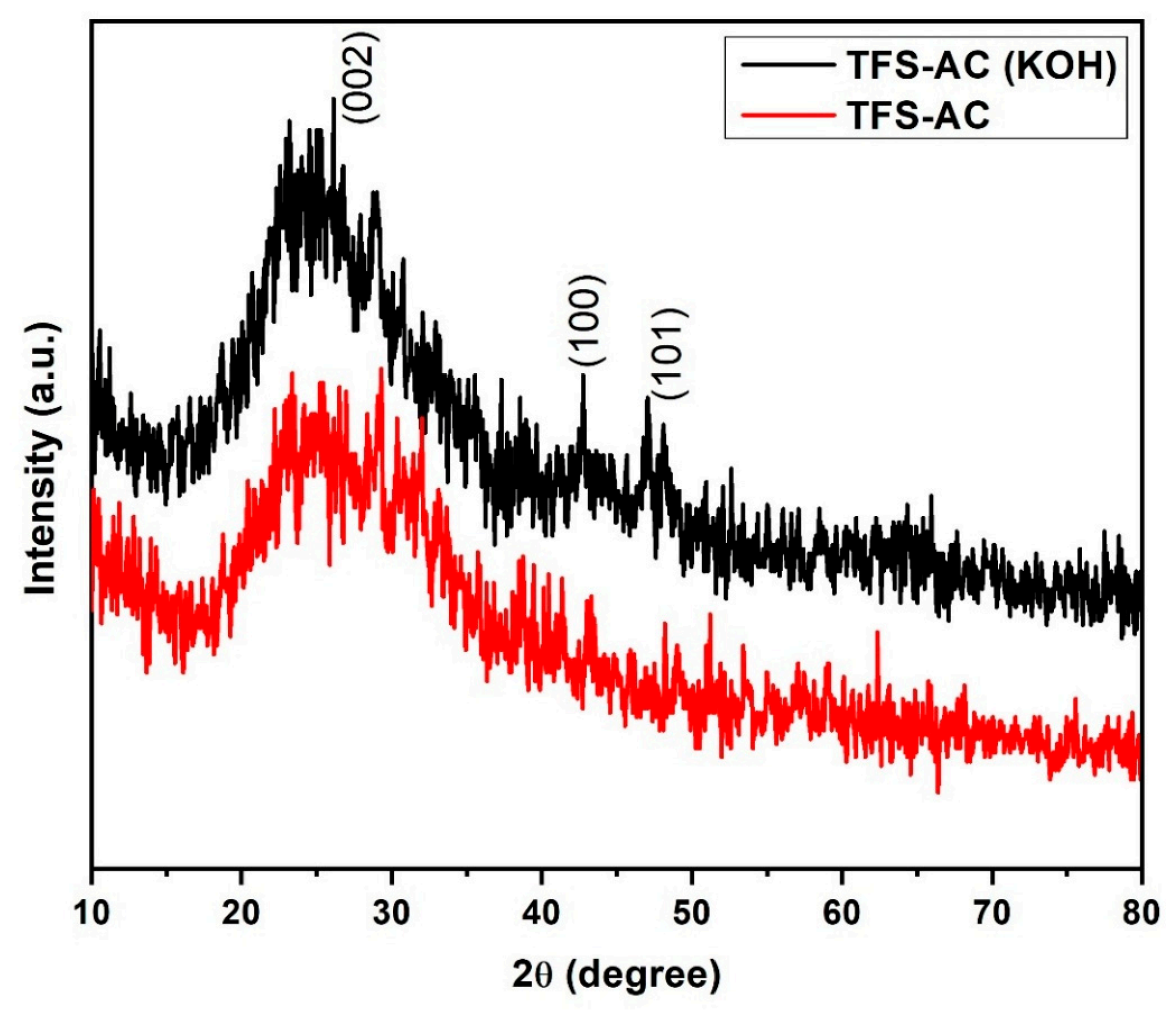
X-ray diffraction (XRD) analysis was carried out for both the pure activated carbon sample (TFS-AC) and the KOH-activated carbon (TFS-AC(KOH)) powder samples.

XRD patterns of the biomass-derived activated carbon in the 2θ range of 10° to 80° to investigate the structural characteristics and crystallinity of the materials. Figure 2 shown in the main diffraction peak for TFS-AC appeared at 2θ = 25.56°, while for TFS-AC (KOH) it slightly shifted to 2θ = 25.02°, both corresponding to the (002) plane of graphitic carbon, indicating the presence of a layered carbon structure with partial graphitization. The broad diffraction peaks observed in both samples suggest a crystalline nature of carbon, commonly seen in biomass-derived carbons due to smaller shifts for KOH activation partially structural disorder. This peak broadening and shift imply increased disorder and expansion of the interlayer spacing after KOH activation, which intercalates the graphitic carbon stacking [38]. Additionally, minor peaks observed in TFS-AC(KOH) at 2θ = 42.65° and 47.10° are indexed to the (100) and (101) planes, respectively, further confirming the development of microcrystalline graphitic domains. Overall, the XRD results demonstrate that the direct activation of tamarind shell-derived carbon via pyrolysis and chemical activation significantly alters the structural order, enhancing the porous nature and crystalline framework beneficial for energy storage applications such as supercapacitors.

### 3.2. RAMAN Analysis

**Figure 3:**
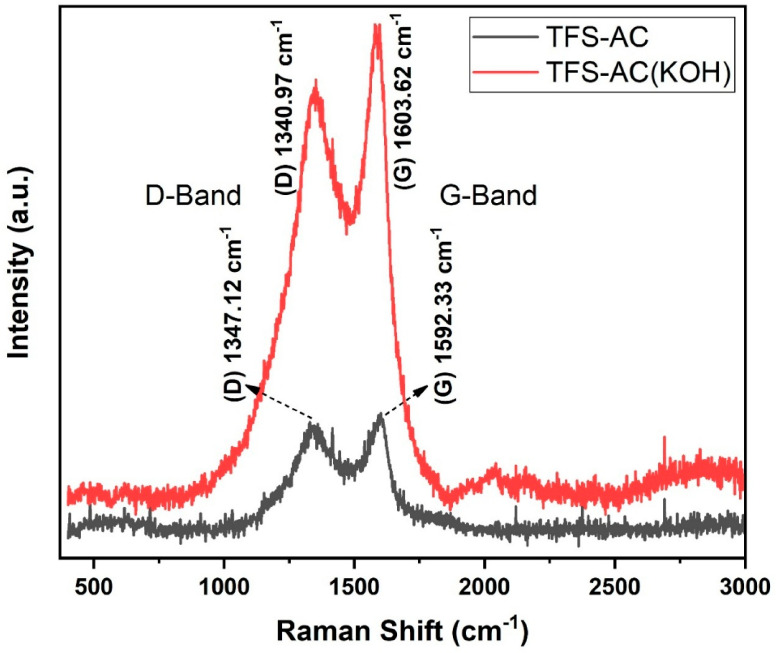
Raman spectra of the activated carbon TFS-AC and TFS-AC(KOH).

Raman spectroscopy is a widely used and sensitive technique for characterizing carbon-based nanostructured materials, as it reveals typical Raman peaks or bands associated with their structural features. The most common Raman bands in crystalline carbon materials are the G-band, corresponding to the graphitic sp^2^-hybridized C–C bonds, and the D-band, which indicates structural defects (Sp^3^) and disorder in the carbon domains. Figure 3 the pure TFS-AC material, the D-band and G-band were observed at 1347.12 cm^−1^ and 1592.33 cm^−1^, respectively, with an intensity ratio (I_D_/I_G_) of 0.82, suggesting a moderate degree of disorder. After KOH activation, the TFS-AC (KOH) sample showed a D-band at 1340.97 cm^−1^ and a G-band at 1603.62 cm^−1^ (Figure 3), with a slightly reduced I_D_/I_G_ ratio of 0.77. This decrease in the intensity ratio indicates a lower level of structural disorder and a higher graphitic character in the activated carbon. The broader D- and G-bands in both TFS-AC and TFS-AC (KOH) further suggest the presence of amorphous or disordered carbon domains. The Raman data confirm that the KOH-activated sample TFS-AC (KOH) exhibits enhanced graphitization and a well-developed porous structure, which are advantageous for charge storage. Furthermore, the presence of highly graphitized, biomass-derived carbon supports efficient ion transport and storage, making the material promising for supercapacitor applications [39,40].

### 3.3. FE-SEM—Morphological Studies

**Figure 4:**
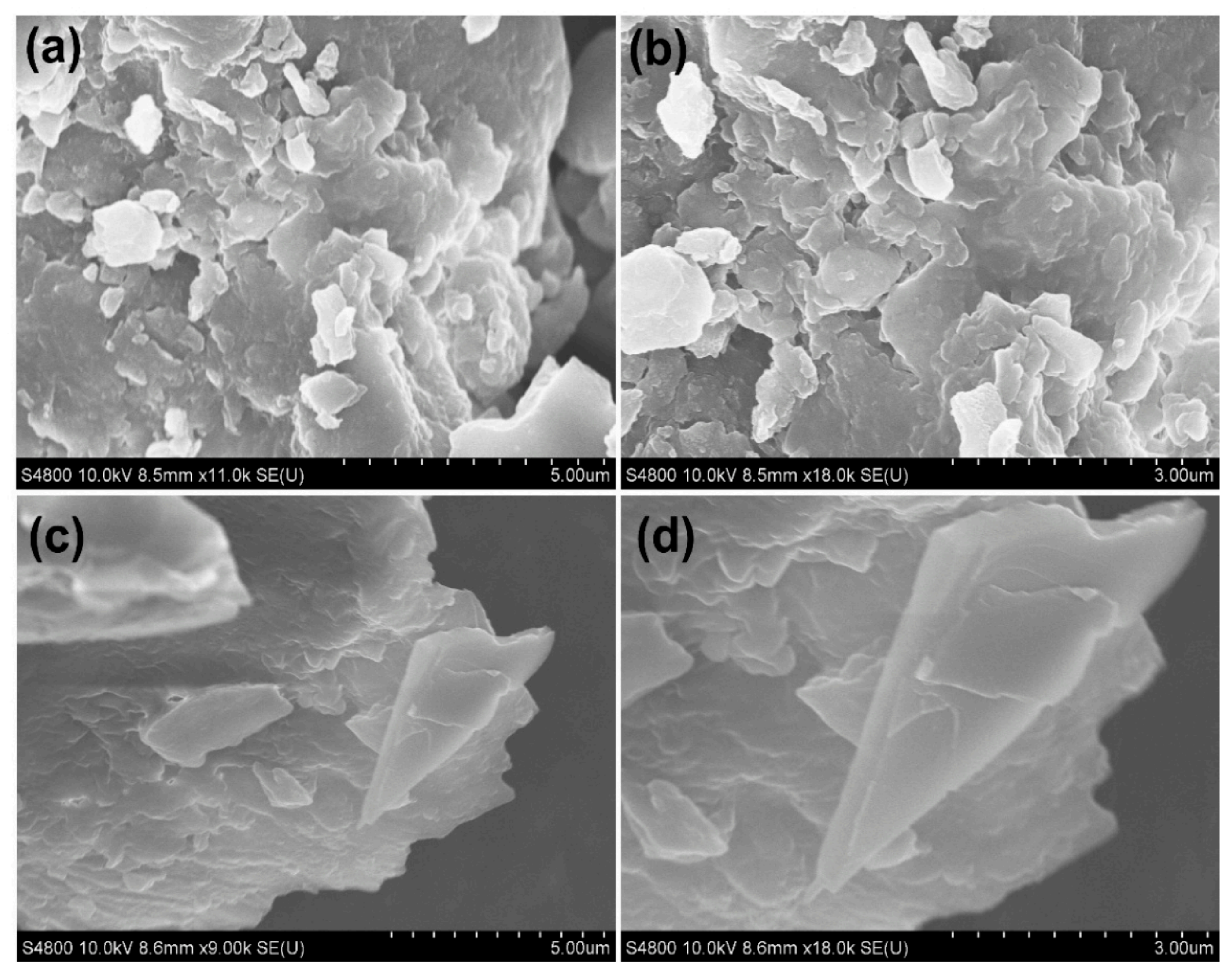
(**a**,**b**) FE-SEM images showing different magnifications of surface morphologies of TFS-AC; (**c**,**d**) TFS-AC (KOH) microscopic images 2D Platelet sheets, layered by interconnected at varying magnifications (5 µm, 3 µm).

The FE-SEM study was conducted to examine the microstructural and morphological characteristics of both TFS-AC and TFS-AC (KOH) samples. As shown in Figure 4a–d, the carbonized TFS-AC sample exhibited a relatively dense and irregular surface morphology with thick, uneven microparticles. In particular, Figure 4a displays multilayered, stacked microparticles with a sheet-like morphology observed under low magnification, indicating a compact, layered carbon structure. In addition, Figure 4b provides a clearer view of horizontally and vertically aligned thick microstructures, similar to skin without KOH activation. These morphological features suggest the presence of overlapping, stacked carbon sheets with different magnifications showing the coexistence of small and thick flattened sheets.

In contrast, the TFS-AC (KOH) sample, which underwent chemical activation with KOH and subsequent pyrolysis, revealed a significantly altered morphology. As shown in Figure 4c, enlarged FE-SEM images illustrate a few layer-like, 2D-layered structures composed of interconnected graphene-like carbon nanosheets. This interconnected porous network is characterized by thin pore walls consisting of just a few layers of carbon. Figure 4d further shows that the carbon platelet microstructure appears foldable and flexible, with a thin-sheet morphology derived from tamarind shell carbon.

Overall, the SEM analysis demonstrates that the un-activated TFS-AC sample primarily consists of stacked, sheet-like carbon layers with minimal or no porosity. In contrast, the KOH-activated TFS-AC (KOH) exhibits a well-developed 3D porous architecture with nanosheet-like, sponge-like features, suggesting the formation of an interconnected carbon framework. The FESEM images in Figure 4c,d clearly confirm the effectiveness of KOH activation and pyrolysis in the facile preparation of an activated carbon sheet-like structure, which is advantageous for electrochemical applications.

### 3.4. FE-TEM—Morphological Studies

**Figure 5:**
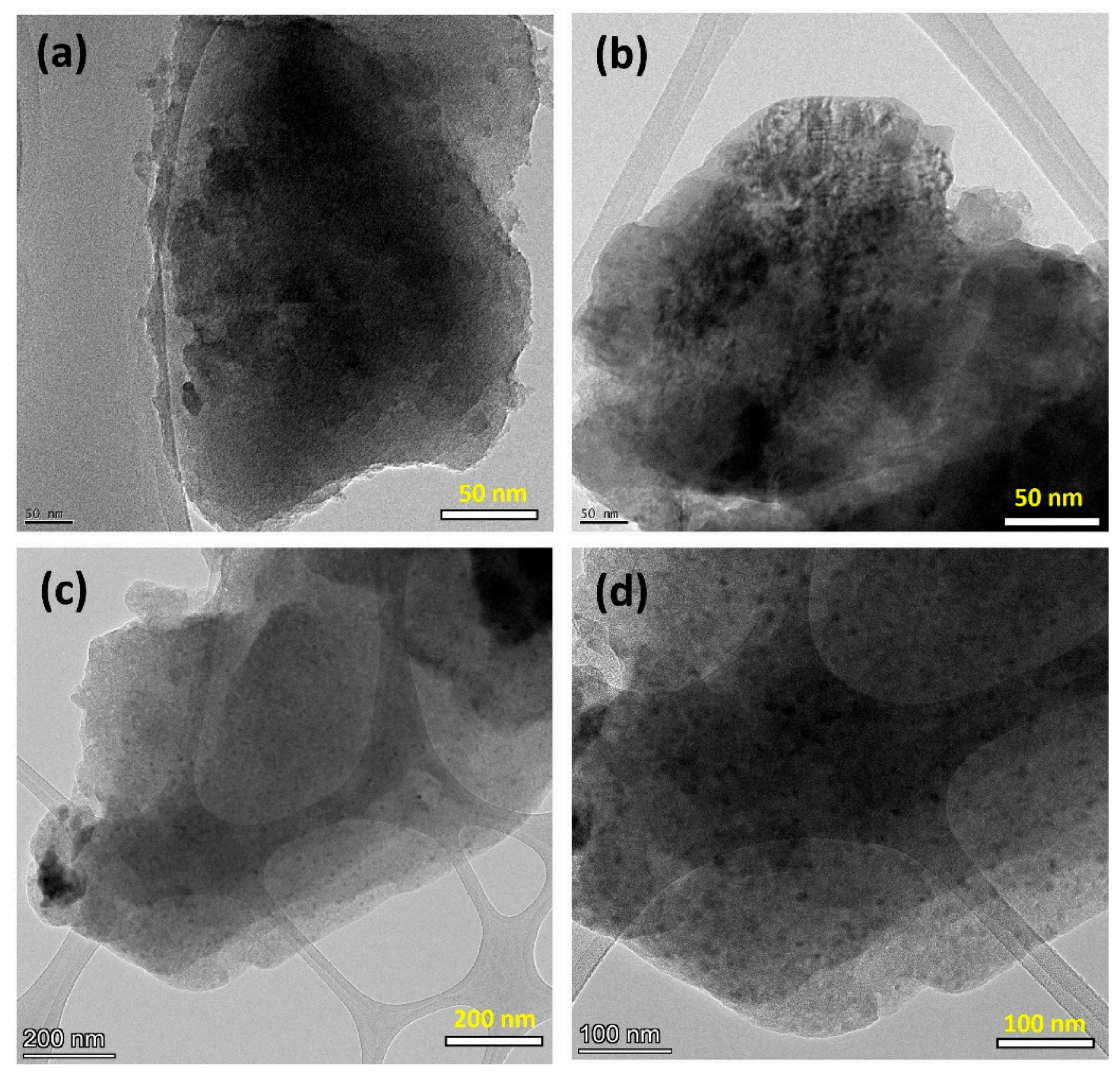
FE-TEM images of (**a**,**b**) pure TFS-AC; (**c**,**d**) KOH-activated TFS-AC(KOH) nano platelets structures.

FE-TEM surface depth analysis was performed to investigate the nanoscale morphology of tamarind-derived activated carbon before and after KOH activation, as shown in Figure 5a–d. The images in Figure 5a display the morphology of pure TFS-AC, revealing dense, thick platelet-like carbon sheets with limited porosity. Figure 5b tamarind seed carbon-stacked layers indicate incomplete exfoliation and less surface area, which may hinder ion transport. In contrast, the KOH-activated sample Figure 5c exhibits a highly fine-edged structure, where few-layer sponge-like regions are clearly visible. These pores result from chemical etching and gas evolution during activation at 800 °C, leading to enhanced surface roughness and thin-layer carbon architectures. The oval-marked region in Figure 5d illustrates exfoliated nanosheets and interconnected few-layer carbon nanosheets. This morphology improves ion diffusion and electrolyte accessibility, which are essential for high-performance supercapacitors. The transformation of tamarind seed outer shell into nanosheet-like porous carbon demonstrates efficient utilization of biomass waste. In addition, KOH activation significantly modifies the surface morphology, creating ultrathin carbon layers with high pore density. This structural enhancement contributes to increased electrochemical surface area and energy storage capacity. Overall, FE-TEM observations confirm that the porous, interconnected nanosheets of TFS-AC (KOH) are ideal for supercapacitor electrode applications.

### 3.5. XPS Analysis

**Figure 6:**
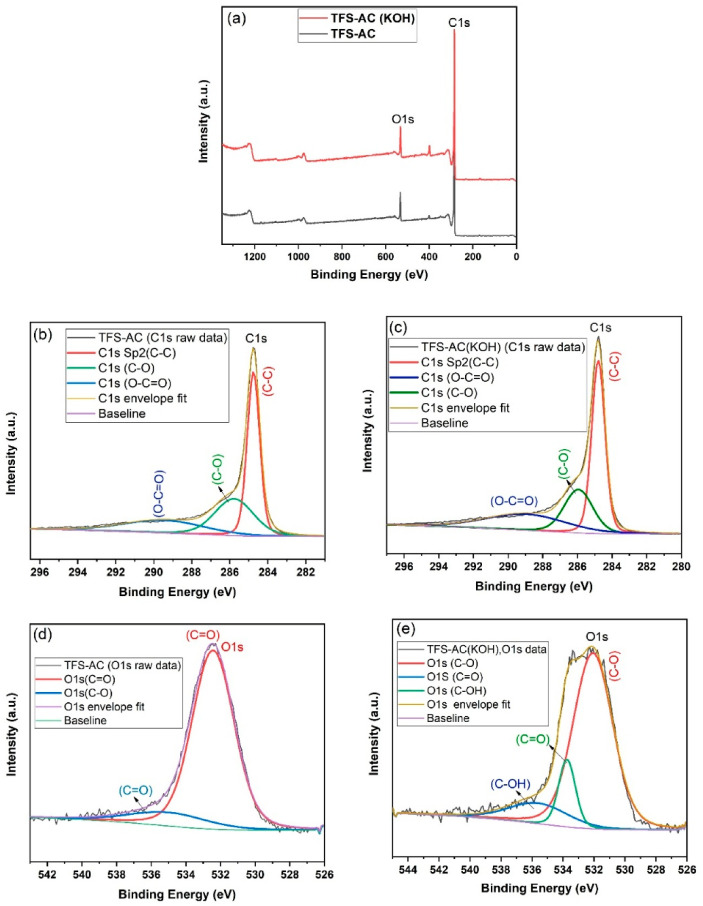
(**a**) XPS survey spectrum about C1s and O1s of tamarind fruit seed shell and derived carbon, the deconvoluted fitted XPS spectra of the TFS-AC and TFS-AC (KOH) samples element fit (**b**) TFS-AC (C1s) and (**c**) TFS-AC(KOH) (C1s), (**d**) TFS-AC (O1s) and (**e**) TFS-AC (KOH) (O1s).

X-ray photoelectron spectroscopy (XPS) was used to investigate the surface elemental composition and chemical bonding states of the TFS-AC and KOH-activated TFS-AC (KOH) samples, as illustrated in Figure 6a–e. The survey spectra in Figure 6a reveal that both samples primarily contain carbon (C1s) and oxygen (O1s) peaks, with significantly enhanced peak intensities for the TFS-AC (KOH) sample, indicating increased surface oxygen functionalities after KOH activation. The C1s high-resolution spectrum of TFS-AC (Figure 6b) is deconvoluted into three peaks at binding energies of 284.75 eV (C–C/Sp^2^ hybridized carbon), 285.77 eV (C–O), and 289.44 eV (O–C=O), confirming the presence of graphitic and oxygen-containing functional groups. After activation, the TFS-AC (KOH) C1s spectrum (Figure 6c) shows similar peaks at 284.79 eV (C–C), 285.93 eV (C–O) and 288.96 eV (O–C=O), but with higher intensity, suggesting that KOH activation introduces more oxygen-rich functionalities and enhances the surface polarity for improved ion accessibility [41].

Figure 6d presents the O1s spectrum for TFS-AC, which is deconvoluted into peaks at 531.23 eV (C=O) and 532.62 eV (C–O), indicating oxygen double and single bonds, respectively. In comparison, Figure 6e for TFS-AC (KOH) shows O1s peaks at 532.06 eV (C–O), 533.74 eV (C=O), and 535.61 eV (C–OH), with a more prominent signal intensity. The presence of –OH groups in the activated sample is attributed to the KOH chemical surface activation process, which introduces hydroxyl-rich defect sites and enhances wettability, thereby improving electrochemical performance. The increase in oxygen content and the formation of multiple fit oxygen (O1s) functional groups support the electrode–electrolyte interface [42]. The C1s/O1s peak intensity ratio is reduced after activation, confirming the relative oxygen enrichment. Overall, XPS analysis demonstrates that KOH activation significantly enhances surface chemistry, leading to improved ion diffusion and storage capability, which are desirable for high-performance supercapacitor electrodes.

### 3.6. Surface Area Analysis by BET Characterization

**Figure 7:**
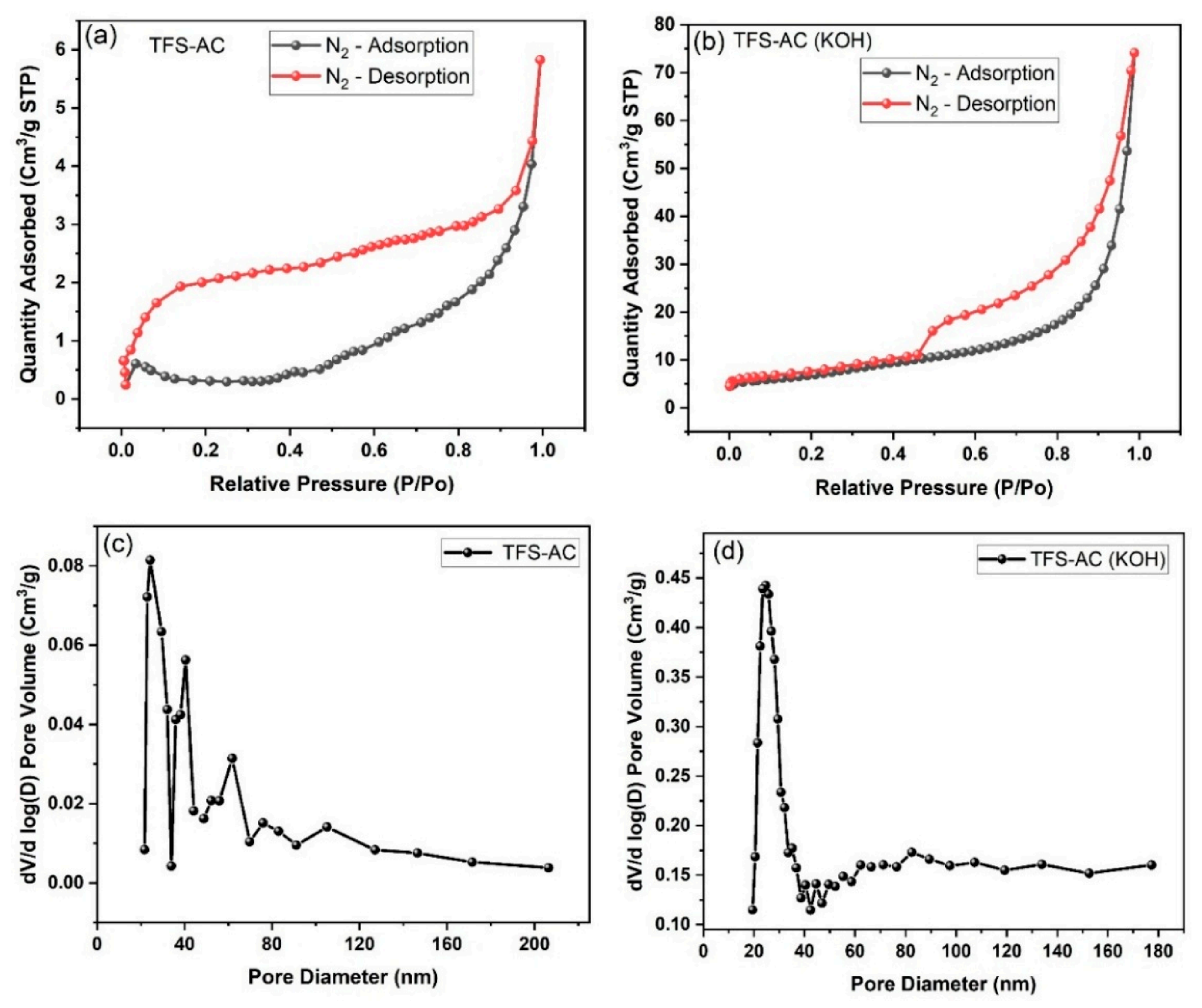
(**a**,**b**) BET N_2_ adsorption–desorption isotherms and surface area analysis for TFS-AC and KOH-activated TFS-AC (KOH) materials (**c**,**d**) pore size distribution plots of and pore volume.

Nitrogen adsorption–desorption isotherms and corresponding pore size distribution plots were used to evaluate the specific surface area, pore volume, and pore size of the TFS-AC and KOH-activated TFS-AC (KOH) samples, as shown in Figure 7a–d. The isotherm of TFS-AC (Figure 7a) shows type-IV behavior with an H3 hysteresis loop, indicating the presence of mesopores with limited pore volume. The BET surface area of TFS-AC is relatively low at 48.54 m^2^/g, with an average pore diameter of 135.64 nm and a total pore volume of 11.79 cm^3^/g, as depicted in the corresponding pore distribution curve (Figure 7c). In contrast, after KOH activation, TFS-AC (KOH) (Figure 7b) displays a sharp rise in nitrogen uptake at high relative pressure (P/P_0_), consistent with microporous-to-nanoporous transition and enhanced pore accessibility. The BET surface area increases significantly to 124.72 m^2^/g, confirming that chemical activation promotes the development of micro- and mesoporous structures. The average pore diameter also increases to 179.52 nm with an enhanced pore volume of 12.69 cm^3^/g, as shown in Figure 7d. This broad pore size distribution and higher porosity indicate the formation of a hierarchical porous structure favorable for electrolyte ion transport and storage [36]. Overall, the KOH activation strategy is highly effective in tuning the porous architecture of TFS-based carbon, making it a promising candidate for future energy storage devices.

## 4. Electrochemical—Supercapacitor Applications

### 4.1. Three-Electrode Cell Analysis

**Figure 8:**
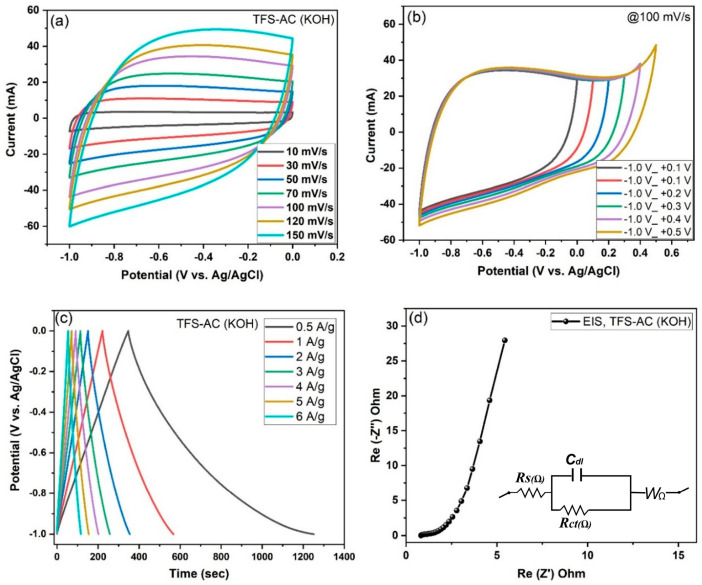
(**a**) CV curve various scan rates for TFS-AC tamarind biomass-derived carbon from TFS-AC (KOH) electrodes, (**b**) CV curves with different potential of carbon at fixed scan rate 100 mV/s (**c**) GCD curves of different current densities (**d**) EIS—Nyquist plot of TFS-AC (KOH) biomass carbon electrodes.

The electrochemical performance of the TFS-AC and KOH-activated TFS-AC (KOH) electrodes was evaluated in a three-electrode system using 3M KOH aqueous electrolyte. The optimized potential window for the negative electrode was determined to be in the range of −1.0 V to 0.0 V (vs. Ag/AgCl), suitable for supercapacitor applications. Cyclic voltammetry (CV) and galvanostatic charge–discharge (GCD) studies were conducted within this potential range to analyze the capacitive behavior of tamarind biomass-derived carbon materials. Various potential windows were initially tested, and an optimal range was selected to ensure maximum charge storage and stable electrochemical response. Electrochemical impedance spectroscopy (EIS) was employed to investigate the resistive and capacitive characteristics of the electrodes. The Nyquist plots show the real part (Z′, x-axis), representing internal and interface resistance, and the imaginary part (Z″, y-axis), indicating capacitive behavior, along with the Warburg region at lower frequencies corresponding to ion diffusion. Additionally, the detailed CV, GCD and EIS responses of TFS-AC and TFS-AC(KOH) electrode data demonstrate Figure 8a–d and Figure S1a–d. The results demonstrate typical ideal electric double-layer capacitor (EDLC) behavior with improved conductivity and ion transport after KOH activation, confirming the enhanced electrochemical performance of the KOH-activated carbon for energy storage applications.

### 4.2. Electrochemical Analysis: TFS-AC

The electrochemical performance of the TFS-AC electrode, derived from tamarind biomass without chemical activation was evaluated in a half-cell configuration using 3M KOH aqueous electrolyte (Supplementary Information Figure S1). Figure S1a shows the cyclic voltammetry (CV) curves at scan rates ranging from 10 to 150 mV/s within the potential window of −1.0 to 0.0 V (vs. Ag/AgCl). The CV curves retain a nearly rectangular shape even at high scan rates, confirming electric double-layer capacitor (EDLC) behavior with absence of faradaic redox peaks, indicative of pure capacitive performance from activated carbon. To further optimize the voltage window, CV was recorded at a fixed scan rate of 100 mV/s in varying voltage ranges (−1.0 to 0.0 V), as shown in Figure S1b. The results demonstrate stable capacitive profiles up to 0.0 V, beyond which increasing potential up to +0.5V potential suggests non-ideal behavior with partial oxygen evaluation. Thus, −1.0 to 0.0 V was identified as the optimum working window for the negative electrode. Figure 8c presents galvanostatic charge–discharge (GCD) curves at current densities from 0.5 to 6 A/g, overall exhibiting symmetrical triangular shapes, confirming high coulombic efficiency and capacitive nature. The Specific capacitance (Csp) values calculated from discharge times were 142.35, 109.77, 90.60, 81.15, 64.68, 43.87 and 29.40 F/g, corresponding to 0.5, 1, 2, 3, 4, 5 and 6 A/g, respectively. These capacitance results decreased values, while increasing current density due to ion diffusion limitations, aligning with CV trends and reinforcing EDLC behavior [43]. Composed, CV and GCD analyses confirm the stable electrochemical performance of TFS-AC as a revealed EDLC performance.

Figure S1d shows the Nyquist plot obtained from electrochemical impedance spectroscopy (EIS), illustrating both internal and interfacial resistances. The impedance plot reveals a Randles electrical circuit consists at the x-axis, indicating a solution resistance (Rs) of 0.86 Ω, while the diameter of the semicircle corresponds to a charge transfer resistance (Rct) of 2.86 Ω, associated with the electrode–electrolyte interface. The linear region at low frequency with a 45° angle reflects the Warburg impedance (W_Ω_), suggesting favorable ion diffusion within the porous structure. These findings confirm that the TFS-AC material, activated at 800 °C, possesses low resistance and efficient ion transport characteristics in electric double-layer capacitance (Cdl). These biomass-derived carbon electrodes have effective wettability for energy storage applications.

### 4.3. Electrochemcial Analysis: Activated TFS-AC (KOH)

The KOH-activated tamarind biomass carbon electrode (TFS-AC (KOH)) was evaluated using a three-electrode system in 3 M KOH electrolyte, revealing significantly enhanced electrochemical properties due to increased porosity and surface area. Figure 8a displays CV curves over scan rates from 10 to 150 mV/s within −1.0 to 0.0 V (vs. Ag/AgCl), showing a nearly rectangular and symmetric profile even at higher scan rates, indicating superior electric double-layer capacitor (EDLC) behavior with rapid ion transport and no redox peaks. The CV profile shows that excellent capacitive response is attributed to the highly porous structure induced by KOH activation, facilitating fast electrolyte ion diffusion and adsorption/desorption kinetics. Figure 8b illustrates the voltage window optimization at a constant scan rate of 100 mV/s, with voltage ranges increasing from −1.0 to +0.5 V. The CV curves remain in a stable positive region, confirming the ideal operational window and capacitive nature. Additionally, GCD curves shown in Figure 8c present well-defined triangular charge–discharge patterns across current densities from 0.5 to 6 A/g, confirming high coulombic efficiency and minimal IR drop. From the GCD curves, the specific capacitance (Csp) values were calculated as 300.34, 244.32, 219.38, 193.36, 160.23, 137.30 and 97.65 F/g for respective current densities of 0.5, 1, 2, 3, 4, 5 and 6 A/g, indicating excellent rate capability and EDLC characteristics. The TFS-AC (KOH)-based maximum specific capacitance values (300.34 F/g at 0.5 A/g) were significantly enhanced due to enhanced pore accessibility and effective ionic charge storage kinetics [44,45]. In addition, as a high-performance supercapacitor electrode, CV and GCD results consistently demonstrate superior electrochemical performance of TFS-AC (KOH). The EIS analysis in Figure 8d shows a Nyquist plot with low solution resistance (Rs) of 0.75 Ω and charge transfer resistance (Rct) of 2.86 Ω, along with a vertical slope in the low-frequency region, indicating highly capacitive behavior and efficient electrolyte ion diffusion pathways. The low Rs and Rct values reflect strong conductivity and minimal interfacial hindrance. These electrochemical evaluations validate that TFS-AC (KOH), activated at 800 °C, exhibits excellent EDLC behavior with high capacitance and low internal resistance, making it a promising candidate for energy storage devices.

The KOH-activated TFS-AC (KOH) electrode exhibited significantly enhanced electrochemical performance compared to the non-activated TFS-AC due to increased surface area and porosity. CV curves for TFS-AC (KOH) showed higher current response and better rectangularity, indicating improved EDLC behavior. In addition, GCD analysis revealed that TFS-AC(KOH) delivered a maximum specific capacitance of 300.34 F/g at 0.5 A/g, compared to twice that of TFS-AC (142.35 F/g). The EIS results showed lower solution resistance (Rs = 0.75 Ω) for TFS-AC (KOH) compared to TFS-AC (Rs = 0.86 Ω), and lower charge transfer resistance (Rct ≈ 2.86 Ω), which is EIS-Nyquist plots, suggesting good ionic transport and electrode–electrolyte interface kinetics [46]. The optimized voltage window and efficient charge storage behavior were more prominent in the KOH-activated carbon platelet electrodes for supercapacitor device applications.

### 4.4. Two-Electrode Symmetric Device: TFS-AC and TFS-AC (KOH)

**Figure 9:**
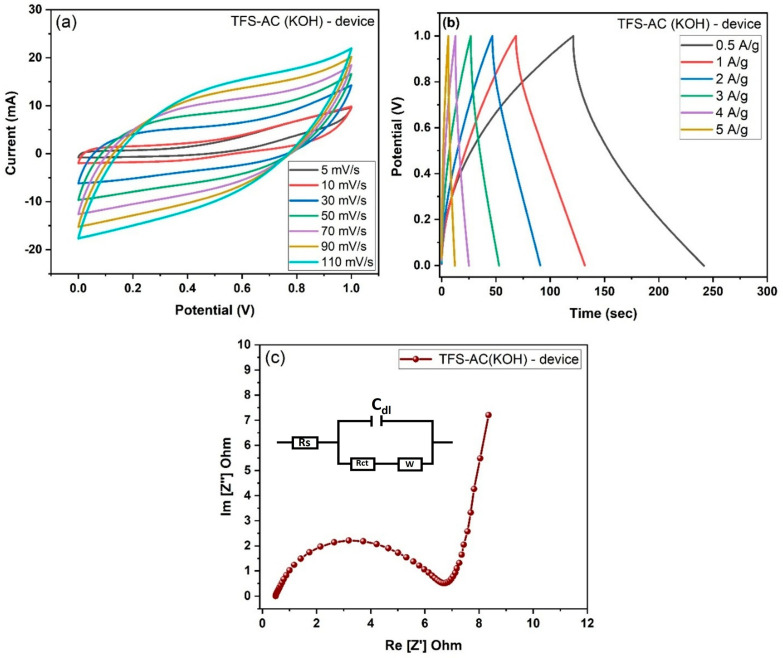
(**a**–**c**): Electrochemical presentation in symmetric device using TFS-AC-KOH electrodes; (**a**) (CV) curves at different scan rates (5–100 mV/s); (**b**) GCD curves at different current densities 0.5–5 A/g; (**c**) EIS-Nyquist impedance plot with Randles circuit (inset).

The symmetric supercapacitor device fabricated using pure TFS-AC (KOH activation) electrodes in Figure 9. The TFS-AC//TFS-AC symmetric device was evaluated in electrochemical data presented in Supplementary File Figure S2). Figure S2a displays the cyclic voltammetry (CV) curves recorded at scan rates ranging from 5 to 100 mV/s within a potential window of 0.0–1.0 V. The CV curves maintain a nearly rectangular shape across all scan rates, signifying ideal electric double-layer capacitor (EDLC) behavior without any redox peaks, which confirms the absence of faradaic reactions due to the non-functionalized nature of the pure carbon. Figure S2a CV demonstrates stable voltage response throughout the 0.0–1.0 V potential range under various scan rates, further supporting excellent capacitive behavior in the symmetric device configuration.

Figure S2b presents galvanostatic charge–discharge (GCD) curves with near-symmetric triangular profiles, indicating good charge–discharge reversibility and coulombic efficiency. The calculated specific capacitance (Csp) values based on GCD discharge times were found to be 78.82, 52.73, 40.51, 27.39, 15.16 and 8.24 F/g at current densities of 0.5, 1, 2, 3, 4 and 5 A/g, respectively, demonstrating a decrease in capacitance with increasing current density due to limited ion diffusion at higher rates. Both CV and GCD results consistently confirm the EDLC characteristics of the symmetric TFS-AC device.

Furthermore, the electrochemical impedance spectroscopy (EIS) analysis, as shown in the Nyquist plot (Figure S2c), reveals a semicircular arc in the high-frequency region and a straight line inclined at approximately 45° in the low-frequency region, characteristic of ideal capacitive behavior. The equivalent circuit consists of Rs-solution resistance, Rct-charge transfer bulk resistance, and Cdl- ideal capacitance of the electric double-layer; the linear slope belongs to Warburg diffusion impedance. In addition, the series resistance (Rs) was measured to be 2.15 Ω, while the charge transfer resistance (Rct) was approximately 14.5 Ω, indicating moderate electrode–electrolyte interface resistance, which is typical for symmetric carbon-based devices. These results confirm the applicability of the pure TFS-AC symmetric device for efficient energy storage applications using electric double-layer charge storage mechanisms.

The symmetric device of pure TFS-AC (KOH)-based activated carbon electrodes was thoroughly investigated through electrochemical techniques such as CV, GCD and EIS to evaluate its supercapacitor performance, as shown in Figure 9a–c. In Figure 9a, the cyclic voltammetry (CV) curves recorded at different scan rates (5, 10, 20, 30, 40 and 50 mV/s) exhibit nearly rectangular shapes without any visible redox peaks, clearly indicating typical electric double-layer capacitor (EDLC) behavior and confirming the absence of faradaic reactions due to the use of pure activated carbon. The CV performance under the potential range of 0–1.0 V further suggests excellent charge propagation and ion diffusion within the electrode material. As shown in Figure 9b, the galvanostatic charge–discharge (GCD) curves display highly symmetric and ideal triangular shapes at various current densities, signifying good reversibility and capacitive efficiency. Based on the GCD profiles, specific capacitance values were calculated using the standard Equation (S1), yielding 129.03, 77.12, 54.93, 41.18, 24.49 and 13.45 F/g at current densities of 0.5, 1, 2, 3, 4 and 5 A/g, respectively. These results reinforce the EDLC characteristics of the device and align well with the CV analysis.

Furthermore, Figure 9c illustrates the (EIS) Nyquist plot, showing a well-defined semicircle in the high-frequency region and a nearly vertical line in the low-frequency region. In addition, the inset Randles circuit consists of Rs (solution resistance), Rct (charge transfer resistance) and Cdl (double-layer capacitance). The real axis intercept reveals a solution resistance (Rs) of 0.49 Ω, while the diameter of the semicircle corresponds to a charge transfer resistance (Rct) of 6.76 Ω, indicating efficient ion transport at the electrode–electrolyte interface. The 45°-inclined line and lower Rs values suggest effective ion diffusion pathways and low internal resistance, confirming the good electrochemical conductivity of the symmetric TFS-AC (KOH)-based device. Thus, the overall results clearly demonstrate the EDLC behavior and potential applicability of tamarind-derived activated carbon for high-performance energy storage in symmetric supercapacitor devices.

The symmetric device using TFS-AC (KOH) electrodes showed significantly enhanced electrochemical performance compared to the pure TFS-AC-based device. At an operating potential of 0.0–1.0 V in 3M KOH aqueous electrolyte, both devices displayed ideal EDLC behavior in CV and GCD analysis. The maximum specific capacitance achieved pure TFS-AC electrodes for symmetric devices was 78.82 F/g at 0.5 A/g, whereas pure TFS-AC electrodes achieved 129.03 F/g at 0.5 A/g. This enhanced specific capacitance result is attributed to the increased surface area and pore accessibility from KOH activation. The KOH-activated carbon also exhibited lower internal resistance and better charge transport, as confirmed by Nyquist impedance plots. Overall, the TFS-AC (KOH)-based device outperformed in overall electrochemical parameters compared to the pure carbon electrodes (TFS-AC) symmetric device.

**Figure 10:**
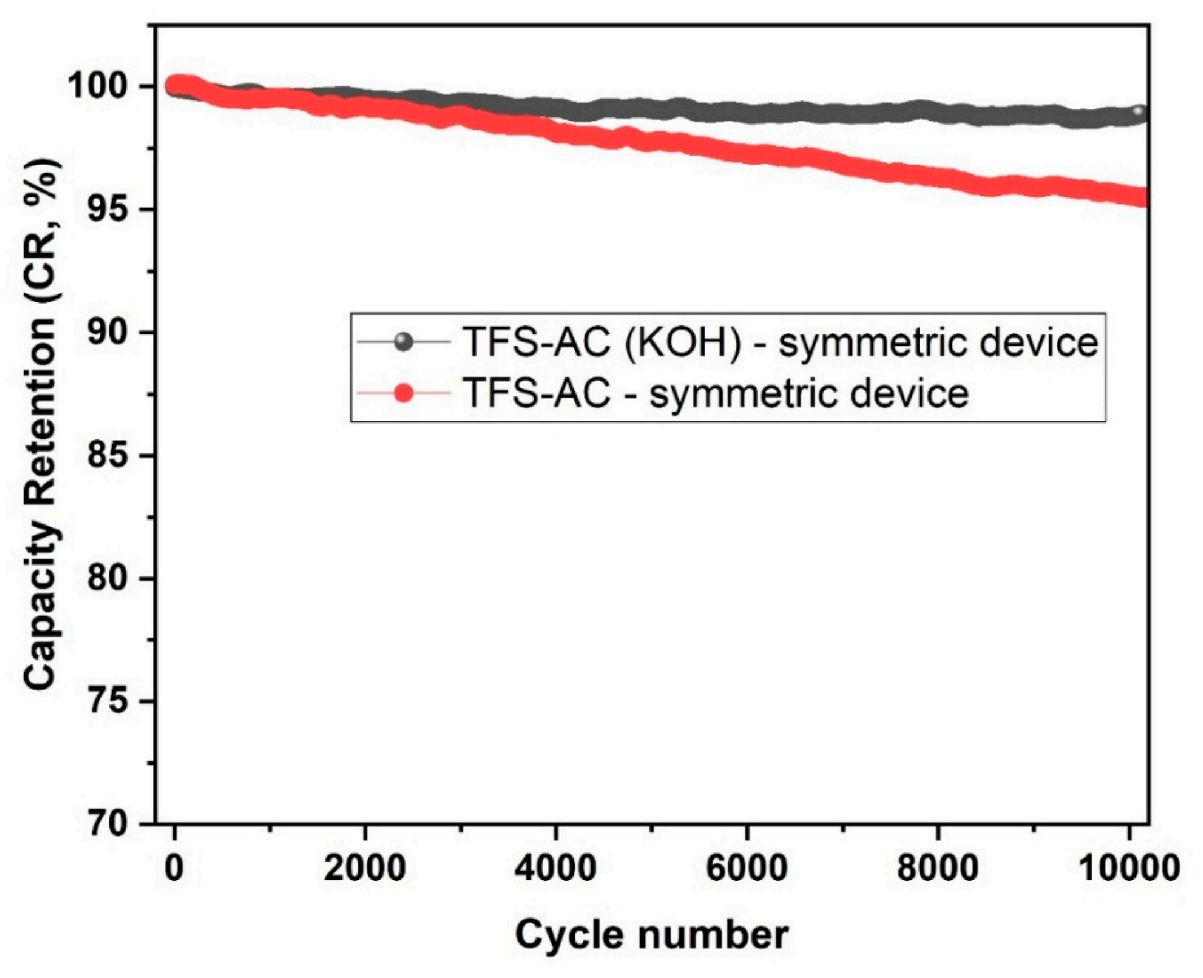
Cycling stability performance of symmetric supercapacitor devices using TFS-AC and TFS-AC (KOH) electrodes over 10,000 capacity retention (CR, %) cycles at 5 A/g.

The cycling stability performance demonstrates the capacity retention (CR%) of TFS-AC (KOH) and TFS-AC electrodes for symmetric supercapacitor devices tested using over 10,000 charge–discharge cycles, as shown in Figure 10. The KOH-activated carbon (TFS-AC (KOH)) exhibits superior electrochemical stability, retaining approximately 98.2% of its initial capacitance, whereas the non-activated TFS-AC device maintains around 95.4%. This enhanced cyclic performance of TFS-AC (KOH) can be attributed to its higher surface area, well-activated few-layer carbon platelet structure, and improved ionic transport kinetics resulting from KOH activation [47].

The gradual capacitance decay observed in the TFS-AC device is likely due to its relatively lower surface area and limited pore accessibility, leading to higher resistance and reduced charge storage efficiency over prolonged cycling. The excellent capacitance retention of TFS-AC (KOH) highlights its good ionic storage and porous structural integrity and long-term durability under repeated cycling, making it a promising electrode material for high-performance and stable energy storage applications.

## 5. Conclusions

A facile preparation of activated carbon from tamarind fruit seed shells (TFSs) was successfully demonstrated, yielding 2D porous carbon platelets. The clean production of graphitic carbon nanosheets of carbonization of TFSs via pyrolysis in an argon (Ar) atmosphere at 800 °C. Both pure TFS-AC and KOH-activated TFS-AC (KOH) revealed highly interconnected porous nanosheet structures with uniform thickness, as observed through morphological analysis. Raman spectroscopy confirmed the graphitic nature of the materials and BET analysis revealed that the TFS-AC (KOH) surface area significantly increased to 124.72 m^2^/g. Finally, electrochemical characterization using CV, GCD and EIS confirmed the potential of both TFS-AC and TFS-AC (KOH) as electrode materials for supercapacitor applications. The as-prepared electrochemical supercapacitor based on tamarind seed bio-mass carbon, applied in half-cell configuration, exhibited a specific capacitance of 300.34 F/g at 0.5 A/g. Finally, compared to the TFS-AC (KOH)// TFS-AC (KOH) symmetric device exhibited a much to 129.03 F/g for the pure TFS-AC device and compared to TFS-AC// TFS-AC device delivered 78.82 F/g at 0.5 A/g. In conclusion, bio-derived KOH-activated carbon platelet nanosheets demonstrated excellent surface area and porosity, lower charge-transfer resistance conductivity enhanced surface area, bio-waste covert into bio-mass products for future energy storage devices.

## Data Availability

This work data are available from the corresponding author upon reasonable request.

## Supplementary Materials

The following supporting information can be downloaded at https://www.mdpi.com/article/10.3390/nano16150957/s1, Figure S1. (a) CV curves various scan rates for carbons derived from TFS-AC material, (b) CV curves at different potential with fixed scan rate 100 mV/s (c) GCD curves of TFS-AC tamarind biomass carbon (d) EIS - Nyquist plot of TFS-AC carbon electrodes and inserted Randles electrical circuit; Figure S2. (a–c): Electrochemical device performance of pure TFS-AC; (a) (CV) curves at different scan rates (5 to 100 mV/s); (b) GCD curves at different current densities; (c) EIS Impedance Nyquist plot with Randles circuit (inset).
