# Supplementary material for: Activation of Biomass-Derived Carbon Platelets for EDLC Symmetrical Devices
Source: Nanomaterials (Basel). 2026 Aug 4;16(15):957. doi: 10.3390/nano16150957 (PMC13468372; doi:10.3390/nano16150957)
Supplement: Supplementary file 1 [file nanomaterials-16-00957-s001.zip › nanomaterials-4472525-supplementary.pdf]

Article: Supplementary File

Activation of Biomass-Derived Carbon Platelets for EDLC Symmetrical Devices

Vediyappan Thirumal[1], Perumal Rajivgandhi[2], Alagan Sekar[2] and Jinho Kim[1]*

[1]Department of Mechanical Engineering, Yeungnam University, Gyeongsan-si, Gyeongbuk-do 38541, Republic of Korea.
[2]Department of Chemistry, Nehru Memorial College (Affiliated to Bharathidasan University), Puthanampatti, Trichy 621 007, Tamilnadu, India.
*Corresponding Authors: jinho@ynu.ac.kr

Supplementary Information (SI)

---

1. Two-electrode, symmetric pouch cell device assembly:

For practical device performance, symmetric pouch cells were fabricated using TFS-AC and TFS-AC(KOH) as the cathode and anode materials, respectively. The electrodes were prepared using the same slurry-casting method as in the three-electrode setup, coated onto nickel foam substrates, and dried at 100 °C. A symmetric configuration was constructed by sandwiching two identical electrodes with a Whatman filter paper separator soaked in 3 M KOH electrolyte. The components were assembled into a flexible pouch-type casing to form a compact symmetric device. Electrochemical testing was conducted in a voltage window of 0 to 1.0 V. Performance analysis included CV, GCD and EIS studies to investigate the capacitance behavior, rate performance and internal resistance of the full device. This setup allowed evaluation of the real-world supercapacitor performance and cycling durability of the bio-derived activated carbon materials.

Both active materials, TFS-AC and TFS-AC (KOH), were coated onto pre-cleaned nickel foam substrates with an active material mass loading of approximately ~1.6–2.8 mg per electrode. After oven drying, the mass loading was determined by measuring the

electrode weight before and after coating. The electrode composition consisted of 80 wt% active material (TFS-AC or TFS-AC (KOH)), 10 wt% conductive carbon, and 10 wt% poly(vinylidene fluoride) (PVDF) binder. The conductive carbon and PVDF binder contributed negligible mass to the electrochemical performance calculations. Based on the measured active material mass, the corresponding specific current was applied in the electrochemical workstation software. The specific capacitance and other supercapacitor performance parameters were calculated using the following equation (Equation S1).

The charge-discharge profile data from these tests were analyzed, and specific capacitance was calculated using the following formula (Eq. (S1)):

$$Csp = \frac{I \times \Delta t}{m \times \Delta V} \text{ F/g} \quad - \quad \text{Eq. - (S1)}$$

Where Eq. (S1) Csp (F/g) is the specific capacitance, I (A) is the charge-discharge current, Δt (s)-discharging time, (m) is the mass of specific active material and ΔV(V)-total potential window.

2. Electrochemical Analysis (supplementary figure):

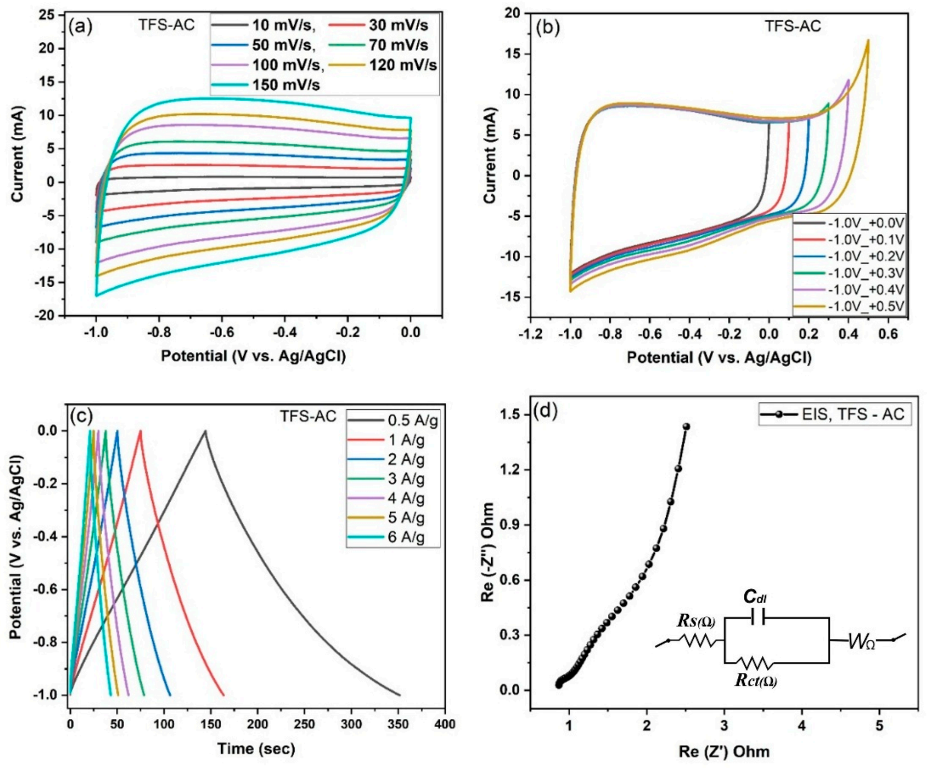

Figure S1. (a) CV curves various scan rates for carbons derived from TFS-AC material, (b) CV curves at different potential with fixed scan rate 100 mV/s (c) GCD curves of TFS-AC tamarind biomass carbon (d) EIS - Nyquist plot of TFS-AC carbon electrodes and inserted Randles electrical circuit.

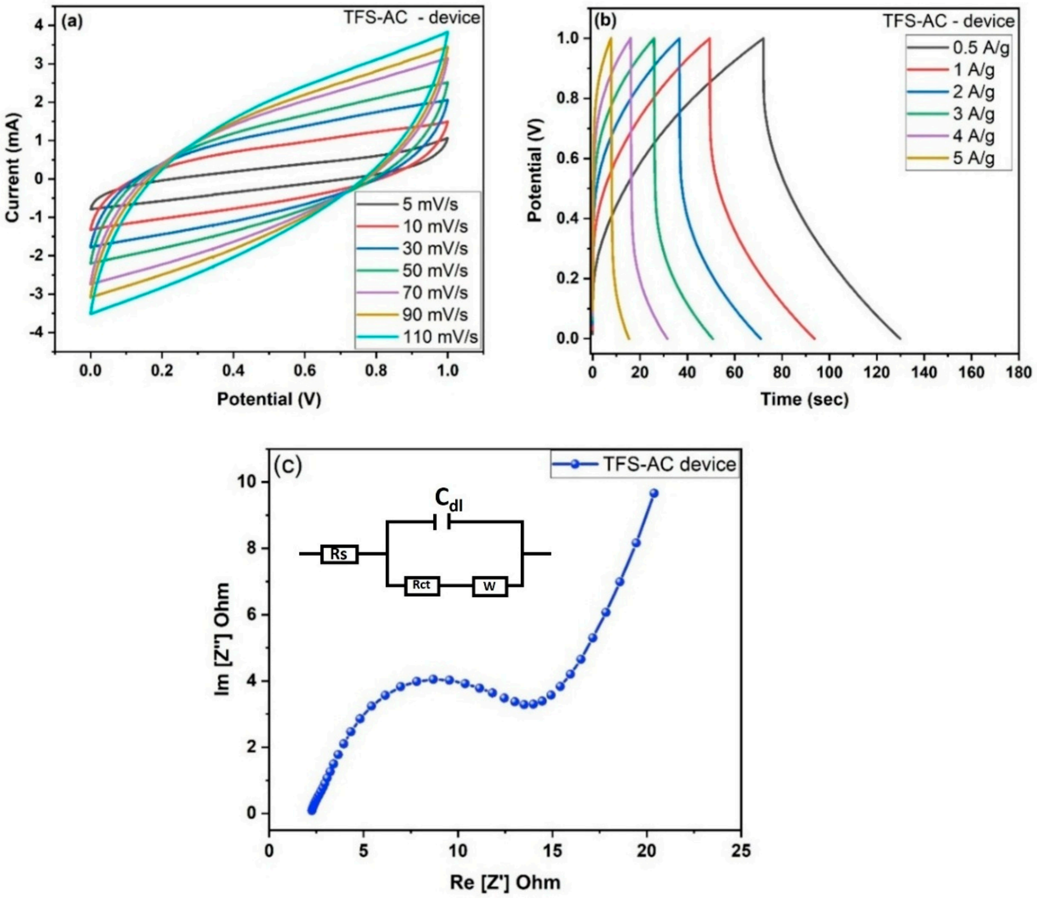

Figure S2. (a–c): Electrochemical device performance of pure TFS-AC; (a) (CV) curves at different scan rates (5 to 100 mV/s); (b) GCD curves at different current densities; (c) EIS Impedance Nyquist plot with Randles circuit (inset).
